# A chronic fatigue syndrome – related proteome in human cerebrospinal fluid

**DOI:** 10.1186/1471-2377-5-22

**Published:** 2005-12-01

**Authors:** James N Baraniuk, Begona Casado, Hilda Maibach, Daniel J Clauw, Lewis K Pannell, Sonja Hess S

**Affiliations:** 1 Georgetown University Proteomics Laboratory, Division of Rheumatology, Immunology & Allergy, Room B-105, Lower Level Kober-Cogan Building, Georgetown University, 3800 Reservoir Road, N.W., Washington DC 20007-2197, USA; 2 Dipartimento di Biochimica A. Castellani, Universita di Pavia, Italy; 3 Center for the Advancement of Clinical Research, The University of Michigan, Ann Arbor, MI, USA; 4 Proteomics and Mass Spectrometry Facility, Cancer Research Institute, University of South Alabama, Mobile, AL, USA; 5 Proteomics and Mass Spectrometry Facility, National Institute of Diabetes and Digestive and Kidney Diseases, National Institutes of Health, Bethesda, Maryland 20892-0508, USA

## Abstract

**Background:**

Chronic Fatigue Syndrome (CFS), Persian Gulf War Illness (PGI), and fibromyalgia are overlapping symptom complexes without objective markers or known pathophysiology. Neurological dysfunction is common. We assessed cerebrospinal fluid to find proteins that were differentially expressed in this CFS-spectrum of illnesses compared to control subjects.

**Methods:**

Cerebrospinal fluid specimens from 10 CFS, 10 PGI, and 10 control subjects (50 μl/subject) were pooled into one sample per group (cohort 1). Cohort 2 of 12 control and 9 CFS subjects had their fluids (200 μl/subject) assessed individually. After trypsin digestion, peptides were analyzed by capillary chromatography, quadrupole-time-of-flight mass spectrometry, peptide sequencing, bioinformatic protein identification, and statistical analysis.

**Results:**

Pooled CFS and PGI samples shared 20 proteins that were not detectable in the pooled control sample (cohort 1 CFS-related proteome). Multilogistic regression analysis (GLM) of cohort 2 detected 10 proteins that were shared by CFS individuals and the cohort 1 CFS-related proteome, but were not detected in control samples. Detection of ≥1 of a select set of 5 CFS-related proteins predicted CFS status with 80% concordance (logistic model). The proteins were α-1-macroglobulin, amyloid precursor-like protein 1, keratin 16, orosomucoid 2 and pigment epithelium-derived factor. Overall, 62 of 115 proteins were newly described.

**Conclusion:**

This pilot study detected an identical set of central nervous system, innate immune and amyloidogenic proteins in cerebrospinal fluids from two independent cohorts of subjects with overlapping CFS, PGI and fibromyalgia. Although syndrome names and definitions were different, the proteome and presumed pathological mechanism(s) may be shared.

## Background

The legitimacy of the diagnosis of Chronic Fatigue Syndrome (CFS) has been questioned because it is a symptom complex without objective markers or known pathophysiology [1]. The 1994 CFS case designation criteria [2] require severe, sudden-onset, disabling fatigue lasting >6 months and ≥ 4 of 8 minor criteria. There are no unequivocal mental, physical, or other aspects of the fatigue that separate CFS from idiopathic, affective or physical illness-related fatigue. The minor criteria can be clustered around complaints of pain (headache, sore muscles, joints, throat, and lymph nodes) and central nervous system dysfunction (concentration/memory difficulties, sleep disturbances, and severe exhaustion after exertion). Many of these symptoms were shared with military personnel from the 1990–1991 Persian Gulf War. Their syndrome was called Persian Gulf War Illness (PGI) [3,4]. Its pathogenesis remains unknown, but likely represented a post-deployment syndrome following the stresses of military hostilities. The term Chronic Multisymptom Illness (CMI) was introduced to describe PGI [4]. Fibromyalgia (FM) is another closely related syndrome, but is characterized by systemic pain and hyperalgesia (tenderness) [5-7]. These sensations implicate dorsal horn and higher central nervous system nuclei that regulate Type C, Aδ and Aβ nociceptive nerve functions.

Patients demonstrate a great deal of overlap between these syndromes. This may not be readily apparent unless a specific effort is made to identify comorbid conditions. Each of the current case designation criteria represents a reductionist approach to focus research on a relatively homogenous group of subjects [1-7]. By necessity this approach may scrutinize only a limited aspect of the complex symptomatology of these patients. The coexistence of syndromes is evidenced by the large number of fibromyalgia subjects who meet criteria for CFS and allied, visceral conditions [8]. For example, dysregulated visceral nociception [9] and mucosal function may lead to an unique nonallergic rhinitis [10-13], noncardiac chest pain, irritable bowel syndrome, vulvodynia, and other "functional" disorders [3,7].

An alternative to the strict reductionist focus on a single syndrome is to thoroughly assess subjects for the wider array of potential co-existing syndromes. This permits a comprehensive and holistic assessment of the broad spectrum of symptom complexes, their presentations, and morphogenesis from one syndrome to another over time. This may provide a more encompassing vision for understanding the basis of these enigmatic conditions.

In this study, groups of subjects were recruited based on the presence of FM, PGI, and no other syndrome (healthy control, HC). When subjects from the FM and PGI groups were assessed more intensively, they were found to simultaneously satisfy the case designation criteria for several of the CFS, PGI, FM, irritable bowel syndrome, and other syndromes. We hypothesized that these subjects had a wide variety of symptom complexes, but that their individual patterns of symptoms were the result of a shared set of underlying pathophysiological molecular mechanisms.

For the sake of discussion in this manuscript, we applied the title "CFS" to the overlapping syndromes afflicting our study group. CFS was the most common individual symptom complex identified in the subjects studied here.

Neurological complaints such as fatigue, pain, affective dysfunction, and dysautonomia were unifying features of these diverse and seemingly unrelated syndromes [1-10]. We hypothesized that central nervous system dysfunction was the primary pathological mechanism that consolidated these syndromes. We predicted that the pattern of cerebrospinal fluid proteins found in CFS would be distinct from control subjects. The differentially expressed CFS – related proteins may be functionally related, and relevant to the pathogenesis of CFS and its allied syndromes. As a null hypothesis, we proposed that CFS, PGI and FM were due to unique pathogenic mechanisms. If so, the patterns of symptoms would vary greatly depending upon the combination of syndromes afflicting any given individual, and there would be no significant differences between cerebrospinal fluid proteomes of the CFS, PGI, FM and healthy control groups.

Our proteomic hypothesis was supported by pilot studies in other disorders. Alzheimer's disease was associated with differential expression of the cerebrospinal fluid proteins cystatin C, two β2-microglobulin isoforms, an unknown 7.7 kDa polypeptide and a 4.8 kDa vascular growth factor polypeptide [14]. Cerebral amyloid angiopathy has been associated with elevated levels of amyloid-β, cystatin C and apolipoprotein E immunoreactive materials [15]. Temporal lobe epilepsy (S100, neuron-specific enolase) [16], and traumatic brain injury (fibrinogen break-down products) [17] were other examples of clinically defined diseases with unique profiles of cerebrospinal fluid proteins (proteomes). We predicted that a pattern of relevant proteins would be found, rather than a single biomarker of disease [14,18-20].

A test – retest study design was used to examine the cerebrospinal fluid proteomes of 2 independent sets (cohorts) of CFS and HC subjects. Different proteomic strategies were used for sample analysis. Cohort 1 consisted of HC, CFS [1,2], and PGI groups [3,4] (n = 10 per group). Cerebrospinal fluids (50 μl/subject) were pooled into single HC, CFS, and PGI specimens. The subjects in the CFS and PGI groups had extensive symptom overlap.

Cerebrospinal fluid samples were examined by reversed phase capillary liquid chromatography – quadrupole – time-of-flight tandem mass spectrometry (CapLC-QToF MS/MS) [21-23]. Proteins were identified and judged to be either qualitatively detectable, or below the lower limits of detection ("absent" or undetectable). The proteins detected in both the pooled CFS and PGI groups, but absent from the pooled HC group, of cohort 1 defined the "cohort 1 pooled CFS proteome". Cohort 1 was equivalent to a "training" set in the test – retest study design.

Cohort 2 consisted of a different group of 12 healthy control and 9 CFS subjects who had their cerebrospinal fluids analyzed individually (200 μl/subject). The individual proteomes of each Cohort 2 subject was obtained. They formed the retest or confirmatory data set for comparison to cohort 1. The individual sets of proteomic results were analyzed by multilinear regression to define those proteins that were detected significantly more frequently in CFS than control subjects. A limited set of proteins was identified as the "cohort 2 CFS-related proteome". Remarkably, these two independent assessments identified 10 proteins shared by the cohort 1 and cohort 2 CFS proteomes and not detectable in healthy control samples.

## Methods

### Subjects

Volunteers meeting criteria for Persian Gulf War illness (PGI) [4], fibromyalgia (FM) [5,6], and healthy control subjects not meeting PGI, FM or CFS [2] criteria were recruited from Georgetown University and Walter Reed Army Hospital Rheumatology and Psychiatry clinics, posters, newspaper and radio advertisements. All subjects gave informed consent. Inclusion criteria included the clinical diagnosis of these syndromes or their absence (healthy controls, HC), and ages of 18 to 60 years. General exclusion criteria were endocrine, allergic, major psychiatric, and other chronic diseases that may account for pain, fatigue or other symptoms; medication use other than stable doses of thyroid hormone; and disorders or lifestyle factors that could markedly affect the hypothalamic – pituitary – adrenal axis or autonomic function (e.g. excessive caffeine, antidepressants, antihypertensive drugs). Medications were discontinued for 3 days prior to study. The protocol and informed consent were extensively reviewed by the Institutional Review Boards of the U.S. Department of the Army, Walter Reed Army Medical Center, and Georgetown University (#1999-090: "Mechanisms of Chronic Multisymptom Illness").

### Subjective and other testing

Subjects were admitted in the evening to the Georgetown University General Clinical Research Center. They were intensively interviewed to identify co-morbid conditions such as CFS [1,2] and irritable bowel syndrome (Rome I criteria) [24,25]. Questionnaires included the Medical Outcomes Survey Short Form 36 (SF-36) [26,27], Multidimensional Fatigue Inventory [28], McGill Pain short form [29], and the Center for Epidemiological StudiesDepression Scale (CES-D) [30,31]. Pain threshold was measured by dolorimetry. The mean of the pressures causing painful sensations at 18 traditional tender points were determined [5,6]. Other aspects of this study, such as standardized measures of hyperalgesia, functional MRI, and autonomic function, will be reported elsewhere.

### Lumbar punctures

At 9 am on the morning after admission, lumbar punctures were performed by a single anesthetist (for consistency). Spinal catheters (23 G) were inserted into the L4–L5 interspace, and 3 tubes of 3 ml of cerebrospinal fluid collected. Fluid from the 3^rd ^tube was centrifuged to remove cells, aliquoted (1 ml), aprotinin (50 KIU per ml) added, and frozen at -80°C until analysis [32]. The bulk of the cerebrospinal fluid was used to measure catechols, other amine neurotransmitters and their metabolites, opioids [33], corticotropin releasing hormone (CRH) [32], neuropeptide Y (NPY) [32], and substance P by ELISA and other methods.

### Cerebrospinal fluid protein preparation

Fluid from 10 HC, 10 PGI and 10 CFS subjects (50 μl/subject) were combined into separate pooled HC, PGI and CFS specimens (cohort 1). At a later date, fluid from 12 HC and 9 CFS subjects (200 μl/subject) were analyzed individually (cohort 2). Proteins were precipitated by adding an equal volume of 100% ethanol, 0.2 N acetic acid, 0.4% sodium bisulfite, chilling at -20°C for 16 hr, followed by centrifugation at 10,000 rpm for 30 min at 4°C [34]. Pellets were resuspended in 50 μl of 0.1 M ammonium bicarbonate pH 7.8, then digested overnight with a 20:1 protein:trypsin ratio (sequence modified grade trypsin, Promega, Madison, WI).

### Liquid chromatography and mass spectrometry

Aliquots (6 μl) of tryptic digests were loaded into the 10 μl loop of a Waters CapLC (Waters Corp., Milford MA USA) [21-23]. Peptides were desalted and concentrated by deposition in a Biobasic C18 precolumn (Thermo Hypersil-Keystone, Bellefonte, PA, USA). They were then eluted through a Zorbax C18 reverse phase column (100 μm × 150 mm, 300 Å particle size, 5 μl volume, Micro-Tech Scientific, Sunnyvale, CA). The buffer gradient began with 95% solution A (0.2% formic acid in water) and increased to 95% solution B (0.2% formic acid in acetonitrile) at 1 μl per min for 100 min. Eluted peptides were ionized by an electrospray device and analyzed by Q-ToF MS/MS (Waters). The total ion current was determined for each mass spectrometry run to confirm that similar amounts of peptides were ionized from each sample.

### Peptide sequencing and protein identification

The "peak list" files (*.pkl files) of raw mass spectra for all peptides were exported into the MASCOT^® ^MS/MS ion search software for protein identification using the NCBInr protein database [35]. The following general search parameters were used: monoisotopic molecular masses, trypsin enzyme specificity, one missed tryptic digestion site, *Homo sapiens *taxonomy, and peptide tolerance of ± 0.4 Da and MS/MS tolerance of ± 0.3 Da. Ions present in many spectra (unknown shared ions and trypsin – autopeptide digest peptides) were excluded [36]. In general, at least 2 peptides covering a minimum sequence of 15 amino acids from unique regions of polypeptides (i.e. not regions of sequence identity shared by other domain or protein family members) were required to identify a single protein [37]. When only a single peptide was identified by MASCOT, manual sequencing was followed by screening against the *Homo sapiens *entries in the NCBInr database [38] using the Protein Information Resource (PIR) PeptideMatch BLAST algorithm [39,40].

### Statistical Analysis

Demographics and other variables were summarized by means and 95% confidence intervals [41,42]. Differences between groups were assessed by ANOVA. If significant (p ≤ 0.05), then differences between individual subsets were assessed by two-tailed, unpaired Student's t-tests with Bonferroni corrections for multiple comparisons.

The lists of qualitatively detected proteins from the 3 pooled HC, CFS and PGI specimens (cohort 1) were compared. Proteins detected in CFS and PGI but not in HC samples were defined as the "cohort 1 pooled CFS proteome".

The individual lists of proteins detected in cohort 2 were compared by multilinear regression (GLM) with stratification by CFS status, gender and age. The proteins significantly associated with CFS status ("cohort 2 CFS proteome") were compared to the "cohort 1 pooled CFS proteome". A step-wise multilogistic model was iteratively created to predict CFS status for cohort 2 with maximum concordance and the minimum number of CFS – related proteins.

Finally, the pooled HC, CFS, and PGI results (cohort 1) were treated as if they were from single individuals, and collated with the cohort 2 results to compare the largest possible number of samples. This was justified because the pooled cohort 1 results were virtually identical to those from the individuals of cohort 2. The frequencies of protein detection between these compiled CFS and HC groups ("all samples") were assessed by ANOVA (differences between means) and Fisher's Exact test (differences in proportions) [41,42].

## Results

### Demographics, pain thresholds and overlapping syndromes

**Figure 1:**
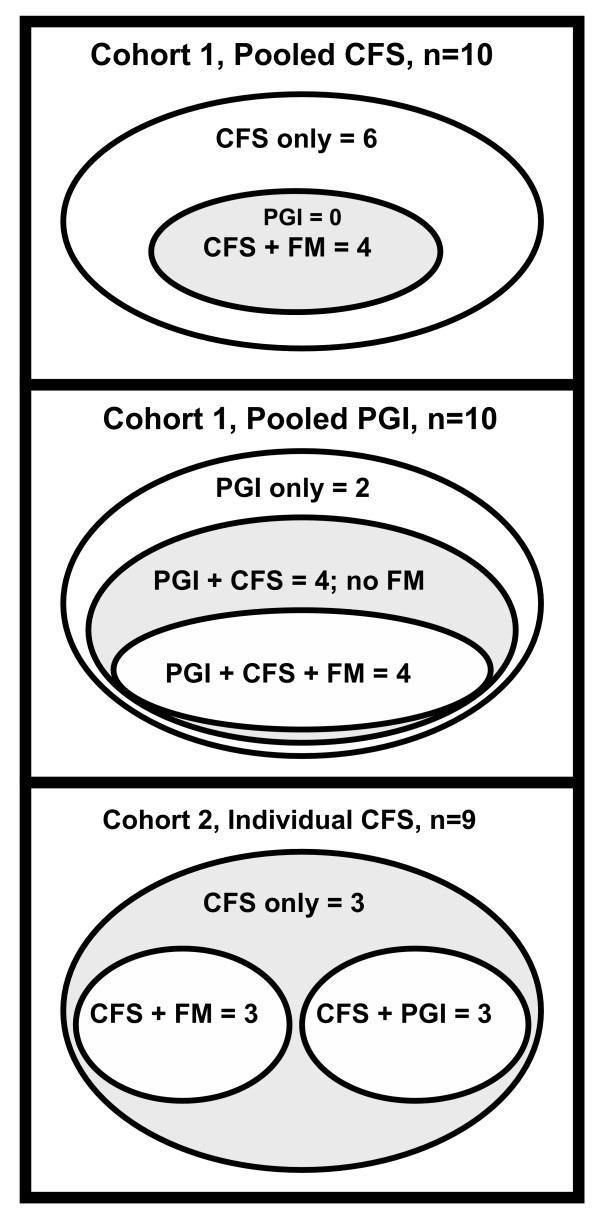
Venn diagram of co-morbid, overlapping syndromes. The numbers of subjects satisfying the case designation criteria for CFS, PGI and FM in the Cohort 1 pooled CFS and pooled PGI groups, and Cohort 2 CFS group are shown. Each group had a highly unique combination of these syndromes.

Ages were not significantly different between groups in either cohort (table 1). There were significantly more males in the pooled HC than pooled CFS group. There was a high degree of overlap of the syndromes of CFS, PGI, and FM within the pooled CFS and pooled PGI groups, and the CFS individuals (figure 1). The cohort 1 pooled CFS group had 4/10 subjects with comorbid FM. The cohort 1 pooled PGI group had 8/10 subjects with comorbid CFS; half of those also had FM. The cohort 2 CFS individuals were divided equally between CFS alone, CFS+FM and CFS+PGI. CFS was present in 27 of 29 subjects, and was the most frequent individual syndrome in cohorts 1 and 2. PGI was present in 13/29 and FM in 11/29. This confirmed that individuals recruited for one syndrome also met case designation criteria for additional syndromes as well.

**Table 1:** Demographics of Cohort 1 (pooled samples) and Cohort 2 (individual samples). Mean (95% C.I.).

**Group**	**N**	**Age (yr)**	**Male**	**CESD**	**Pain Threshold (kg)**
**COHORT 1 (Pooled Samples)**					

**HC Pool**	10	34.4 (29.1 to 39.7)	80%	4.3 (0.6 to 7.9)	7.69 (5.72 to 9.65)
**CFS Pool**	10	39.9 (34.3 to 45.5)	20% ***	17.6 *** (12.1 to 23.0)	4.01 ** (2.86 to 5.16)
**PGI Pool**	10	43.5 (38.7 to 48.3)	60%	18.1 ** (8.7 to 27.5)	4.89 * (3.64 to 6.14)

**COHORT 2 (Individual Samples)**					

**HC**	12	41.3 (33.6 to 48.9)	75%	-	7.17 (5.71 to 8.64)
**CFS**	9	39.1 (32.2 to 46.0)	33%	-	4.97 ^§ ^(3.75 to 6.19)

* p < 0.05, ** p < 0.01, *** p < 0.001 compared to HC Pool results; ^§ ^p < 0.05 compared to HC individuals; ANOVA followed by Student's t-tests.

### SF-36 scores

**Figure 2:**
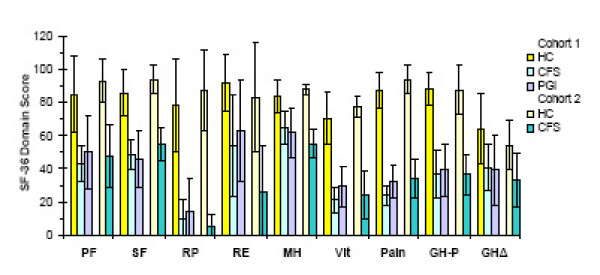
SF-36 scores for each group (mean ± 95% C.I.). Physical Function (PF), Social Function (SF), Role Physical (RP), Role Emotional (RE), Mental Health (MH), Vitality (Vit), Pain, General Health Perception (GH-P) and General Health Change (GHΔ) were identical for the set of pooled (Cohort 1; yellow bars) and individual (Cohort 2; beige bars) HC subjects. These domains were also identical for the set of pooled CFS (light blue bars), pooled PGI (light purple bars) and CFS individuals (teal bars). Significant differences between these datasets were found for all indicators except RE, MH and GPΔ (p < 0.02 by ANOVA).

The HC pooled group and HC individuals gave identical results for all categories, indicating normal quality of life (scores near 100) (figure 2). The pooled CFS and PGI groups, and CFS individuals also had equivalent scores for each domain. Their scores for most domains were significantly different from the respective HC scores (p < 0.02 by ANOVA; p < 0.02 by Student's t-tests after Bonferroni corrections). Exceptions were role emotional (RE), mental health (MH) and general health change (GHΔ) [26,27].

### Multidimensional fatigue inventory scores

**Figure 3:**
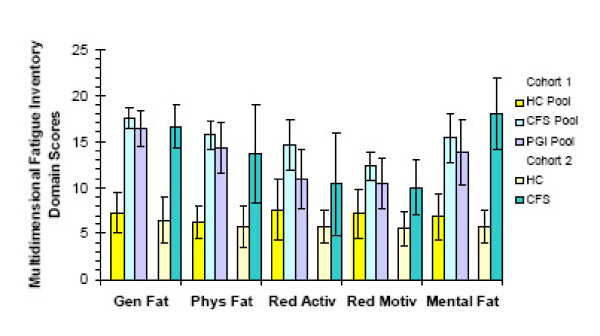
Multidimensional fatigue inventory scores (mean ± 95% C.I.). The healthy control (HC) pooled group (yellow  bars) and individuals (beige bars) had lower scores for all categories than the pooled CFS (light blue bars), pooled PGI (light purple bars), and CFS individuals (teal bars) (p < 0.03 by ANOVA). The categories, from left to right, were general fatigue, physical fatigue, reduced activity, reduced motivation, and mental fatigue.

The pooled HC and HC individuals had lower scores that trended towards normality for all domains (figure 3) [28]. Cohort 1 pooled CFS and pooled PGI scores for all domains were significantly higher than pooled HC (p < 0.01 by t-tests). Pooled PGI had significantly higher scores than pooled HC for general (p = 0.0001), physical (p = 0.006) and mental fatigue (p = 0.02). Cohort 2 CFS individuals had significantly higher scores than HC individuals for general and mental fatigue (p < 0.003).

### Center for epidemiologic studies depression scale (CESD)

Scores over 16 suggest affective dysfunction [30,31]. Questionnaires were completed by the cohort 1 subjects only. Pooled HC had normal, low scores (table 1). Scores for the pooled CFS and CMI groups were similar to each other, and significantly higher than HC.

### Pain and tenderness

The McGill short form pain questionnaire was completed by only 12 HC, 12 CFS and the 10 pooled PGI subjects (table 1). Scores for CFS and PGI groups were significantly higher than HC. Affective scores were 0.1 (-0.1 to 0.2) for HC, 2.5 (1.6 to 3.4) for CFS, and 3.2 (1.2 to 5.2) for PGI (p = 0.002 by ANOVA) [29]. Sensory scores had the same trend: 1.0 (-0.3 to 2.3), 10.2 (6.4 to 13.9) and 10.2 (5.7 to 14.7), respectively (p = 0.0005 by ANOVA). Pressure-induced pain thresholds were similar for both of the HC groups (table 1). The pooled CFS, pooled PGI, and cohort 2 CFS individuals had significantly lower thresholds than their respective controls. This was consistent with the admixture of FM subjects who have sensory hyperalgesia ("tenderness") and pain complaints [5,6].

### Irritable bowel syndrome

About half of all the CFS and PGI subjects had irritable bowel syndrome (Rome I criteria) [24] compared to none of the HC (p = 0.002 by ANOVA).

### Clinical summary

The pooled HC and HC individuals had virtually identical demographic, pain threshold, and psychometric results. The variances for the pooled HC results were small indicating homogeneity regardless of age or gender. Results for cohort 1 CFS and PGI, and cohort 2 CFS subjects were essentially equivalent. Scores were generally significantly different from their HC comparison groups. There was a surprising similarity even though the proportions of subjects with each case designation criteria in each cohort were different. This supported our hypothesis that the designation of CFS, PGI or FM was overruled by the overlapping nature of these syndromes, and that a shared mechanism may have been present. Comparisons of the cerebrospinal fluid proteomes tested this hypothesis.

### Proteomics of cohort 1

Mass spectrometry and bioinformatics analysis identified 17 proteins in the pooled HC group, 63 in the pooled CFS and 40 in the pooled PGI samples (table 2). A total of 73 unique proteins were identified. Twelve were detected in all three pools. The pooled CFS and PGI samples shared 20 proteins that were not detected in the pooled HC group. This formed the "cohort 1 pooled CFS proteome" (table 3, column 2). The most pertinent of these matched proteins had probable origins in plasma or the central nervous system. Probable plasma proteins were the antiproteases α-2-macroglobulin (α-2-mac) and α-1-antichymotrypsin; the metal transport and antioxidant proteins ceruloplasmin (ferroxidase II), haptoglobin and orosomucoid 2 (ORM2; α1-acid glycoprotein, type 2); and complement factors C3 and the C4A and C4B gene products. Brain – derived proteins included amyloid β (A4) precursor like protein 1 (APLP1), autotaxin (ENNP2, ectonucleotide pyrophosphatase/phosphodiesterase 2, phosphodiesterase I/nucleotide pyrophosphatase 2; alkaline phosphodiesterase I), carnosine dipeptidase 1 (CNDP1, carnosinase 1, CN1), and pigment epithelium-derived factor (PEDF). BEHAB (**b**rain **e**nriched **h**y**a**luronan **b**inding protein, brain specific chondroitin sulfate proteoglycan, brevican) and gelsolin were structural proteins. The numbers of proteins detected in each of the 3 pooled sample were proportional to the total ion current for HC (7,000), PGI (26,000) and CFS (33,600) (R^2 ^= 0.94). This raised the possibility that there was less protein in the pooled HC sample or a smaller amount of tryptic peptides were loaded during mass spectrometry than for the pooled CFS and CFS and PGI specimens. These possibilities were examined in cohort 2.

**Table 2:** Yields of proteins identified in each cohort and group. A total of 73 proteins were identified from the 3 pooled HC, CFS and PGI specimens of cohort 1. Twenty proteins were shared by the CFS and PGI specimens that were absent from the pooled HC sample. This was the cohort 1 pooled CFS – related proteome. Cohort 2 consisted of 12 HC and 9 CFS individuals. A total of 113 proteins were identified, with 16 identified by multilinear regression (general linear model, GLM) as the cohort 2 CFS – related proteome. When the proteins from all of the specimens were examined, a set of 19 out of 115 proteins were significantly different between HC and CFS by ANOVA.

	Cohort 1 (pooled samples)			Cohort 2 (individual samples)		All specimens	
Protein yield per cohort	73			113		115	

Groups in each cohort	HC	CFS	PGI	HC	CFS	HC	CFC

Samples per group	1	1	1	12	9	13	11
Proteins per group	17	63	40	71	82	72	83
Proteins shared within cohorts	12	12	12	39	39	40	40
Proteins unique to each group	2	28	8	32	43	32	43
CFS-related proteomes		20			16		19
Statistical method	Matching			GLM		ANOVA	

**Table 3:** Chronic fatigue syndrome – associated cerebrospinal fluid proteomes. Proteins from the cohort 1 – and cohort 2 – related CFS proteomes (+) or not part of these proteomes (0) were shown in columns 2 and 3. The 5 proteins from cohort 2 that were most predictive of CFS (80% concordance) were defined by the "biosignature variable"*B*_*1/5*_(*right justified *and *bold italics*). The 2 cohorts were combined (n = 24 samples) and the frequencies of detection in all control (HC, n = 13) and CFS (n = 11) samples compared by ANOVA and Fisher's Exact Test. NCBI accession numbers (gi|) were given.

	**Cohort**		**CFS – Associated Proteome **(all 24 samples)	HC % (n = 13)	CFS % (n = 11)	ANOVA	Fisher's Exact Test
GeneID							
	**1**	**2**					
720, 721	+	+	Complement C4A/B	0%	55%	0.0010	0.0034
2934	+	+	Gelsolin	0%	45%	0.0046	0.011
3868	**0**	+	***Keratin 16 (B***_***1/5***_)	0%	45%	0.0046	0.011
5176	+	+	***PEDF (B***_***1/5***_)	0%	45%	0.0046	0.011
3537	**0**	**0**	Ig λ	15%	64%	0.014	n.s.
351	+	+	APLP1	0%	36%	0.016	0.031
1356	+	+	Ceruloplasmin	0%	36%	0.016	0.031
2	+	+	***α-2-Macroglobulin (B***_***1/5***_)	0%	36%	0.016	0.031
5005	+	+	***Orosomucoid 2 (B***_***1/5***_)	0%	36%	0.016	0.031
5168	+	+	Autotaxin	0%	36%	0.016	0.031
1114	**0**	**0**	Chromogranin B	0%	36%	0.016	0.031
63827	+	+	***BEHAB (B***_***1/5***_)	0%	36%	0.016	0.031
12	+	**0**	α-1-Antichymotrypsin	8%	45%	0.034	n.s.
3240	+	**0**	Haptoglobin	15%	55%	0.044	n.s.
3263	**0**	**0**	Hemopexin	15%	55%	0.044	n.s.
6696	**0**	**0**	Secreted phosphoprotein 1	0%	27%	0.046	n.s.
3872	**0**	+	Keratin 17	0%	27%	0.046	n.s.
140446	**0**	+	Keratin 6C	0%	27%	0.046	n.s.
718	+	**0**	Complement C3	23%	64%	0.047	n.s.

### Proteomics of cohort 2

The 21 individual samples from cohort 2 contained 113 distinct proteins (table 2). The HC individuals had a cumulative total of 71 proteins, including 32 found only in this group. The 12 HC samples contained an average of 16.3 (14.4 to 18.3), and generated an average total ion current of 17,800 (13,500 to 23,400). There were 39 proteins shared between the two groups in this cohort. CFS subjects had a total of 82 proteins identified. There were 43 proteins found only in CFS. The 9 CFS samples had an average of 23.3 (12.6 to 34.0) proteins per subject, and total ion current of 21,200 (12,800 to 34,900). Since the numbers of proteins detected and total ion currents were not different between the HC and CFS samples, it was likely that the cerebrospinal fluid total protein concentrations were similar. The low number of proteins detected for HC in cohort 1 may have been due to dilution during sample mixing, trypsin digestion or loading for mass spectrometry.

Multilinear regression by general linear modeling (GLM) identified 16 proteins from the cohort 2 CFS samples that were not detectable in the individual HC samples (table 4). Ten of these sixteen proteins were also detected in the "cohort 1 pooled CFS proteome" (table 3). This degree of protein matching between two independent populations of CFS subjects was highly unlikely to be a random event (odds <10^-15^).

**Table 4:** Multilinear regression (general linear model; GLM) analysis of the cohort 2 CFS – associated proteome. Proteins that were detected significantly more frequently in CFS than HC were shown in column 2. The significance (P), explained variance (R^2^), and probabilities for the association of each protein when stratified by CFS status, gender, and their cross-product were tabulated. The presence of at least 1 of the 5 CFS proteome proteins (*right justified *and *bold italics*) was sufficient to identify all the CFS subjects in Cohort 2 (*B*_*1/5*_; see Results). (n.s., not significant).

	**Cohort 2: Multilinear Analysis (GLM; individual samples)**					
	
**GeneID**	Cohort 2 CFS Proteome	**P**	**R**^**2**^	**CFS**	**Gender**	**CFS × Gender**
3868	***Keratin 16 (B***_***1/5***_***)***	0.0001	1	0.0001	0.0001	0.0001
2	***α2-Macroglobulin (B***_***1/5***_***)***	0.001	0.63	0.009	0.023	0.003
1356	Ceruloplasmin	0.001	0.63	0.009	0.023	0.003
5005	***Orosomucoid 2 (B***_***1/5***_***)***	0.001	0.63	0.009	0.023	0.003
5168	***Autotaxin (B***_***1/5***_***)***	0.001	0.63	0.009	0.023	0.003
351	APLP1	0.001	0.63	0.009	0.023	0.003
63827	BEHAB	0.001	0.63	0.009	0.023	0.003
140446	Keratin 6C	0.001	0.63	0.009	0.023	0.003
3872	Keratin 17	0.001	0.63	0.009	0.023	0.003
5004	Orosomucoid 1	0.001	0.63	0.009	0.023	0.003
3858	Keratin 10	0.013	0.48	n.s.	0.016	n.s.
721	C4B	0.022	0.44	0.007	n.s.	n.s.
5176	***PEDF (B***_***1/5***_***) ***	0.022	0.44	0.007	n.s.	n.s.
2934	Gelsolin	0.033	0.41	n.s.	n.s.	0.011
84735	CNDP 1	0.037	0.40	n.s.	0.033	n.s.
3861	Keratin 14	0.037	0.40	n.s.	0.033	n.s.

**Figure 4:**
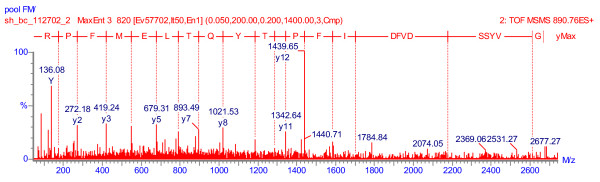
Ceruloplasmin (ferroxidase II) peptide mass spectrogram. This sequencing data was shown for the time-of-flight mass spectrometer (ToF, 2^nd ^MS). The relative signal intensities for each fragment of the ceruloplasmin peptide (y-axis) were plotted against mass/charge (*m/z*; x-axis). The peptide, GVYSSDVFDIFPGTYQTLEMFPR, was sequenced from the y-series (right to left; N-terminal to C-terminal). It had *m/z *= 890.41 and z = 3+, for a mass of 2671.23.

α2-Mac, ceruloplasmin (figure 4), ORM2, and autotaxin were significantly associated with CFS status and gender (table 4). These were also present in the cohort 1 pooled CFS proteome. APLP1, BEHAB, orosomucoid 1 (ORM1, α1-acid glycoprotein, type 1), and keratins 6C and 17 were also associated with CFS and gender. Keratins 16 (K16), 14 and 10 were associated with male gender (table 4). Other gender-related factors may be found in future surveys given the female predominance (80%) of CFS [1,2].

PEDF and C4 were significantly associated with CFS but not to gender (table 4). C4 has 2 genes, C4A and C4B, with the latter more significantly associated with CFS. Gelsolin was associated with CFS status. CNDP1 was originally identified as glutamate carboxypeptidase-like protein 1, but was correctly assigned using PIR website tools [39,40]. All of these proteins were detected in both the cohort 1 pooled CFS and cohort 2 CFS proteomes.

### Predictive statistical model for CFS from cohort 2

Multilogistic analysis demonstrated that a smaller set of 5 proteins could identify all of the cohort 2 CFS subjects. They were α-2-mac, APLP1, K16, OMD2, and PEDF. Subjects who had detectable levels of at least 1 out of these 5 proteins had an odds ratio of 34.5 in favor of having CFS regardless of gender (1.49 to 809.61; p = 0.0072, Fisher's Exact test). This new "biosignature" variable of having ≥1 out of 5 proteins present in the cerebrospinal fluid was defined as ***B***_***1/5***_. It was included in a logistic model to predict CFS status for the 21 subjects in cohort 2:

CFS status = gender + (***B***_***1/5***_)

The model was significant (converged asymptotically) and had a concordance rate of 80%. To our knowledge, this is the first model to predict CFS status based solely on objective data.

### Factor analysis of cohort 2

The syndromic designations, results of the questionnaire and psychometric tests, and components of the proteomic studies were analyzed. No significant factors were identified. This suggested that the cerebrospinal fluid proteome was independent of the exact spectrum of complaints and self-reported symptoms found in the CFS spectrum of illnesses (figure 1). The CFS – associated proteome and ***B***_***1/5 ***_were consistent with our hypothesis that a common pathological mechanism was shared by these allied syndromes, and was independent of the set of symptoms expressed by each individual.

### Combination of all samples

**Figure 5:**
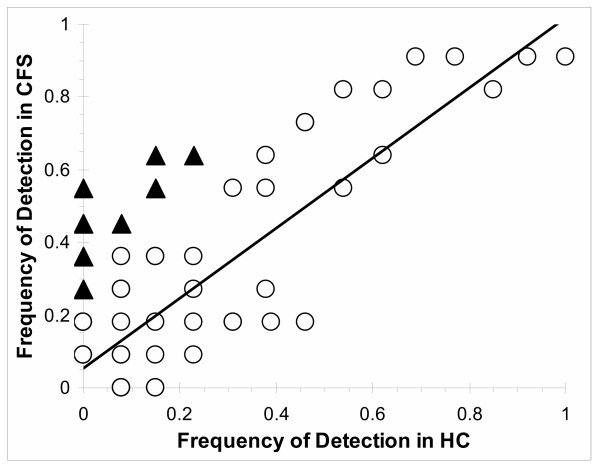
The correlation between the frequencies of protein detection in the CFS (black triangles) and HC (open circles) groups were shown. Nineteen proteins were detected significantly more frequently in the CFS than HC group (p ≤ 0.05 by ANOVA). These CFS – associated proteins were shifted away from the line of identity. This line demonstrated the high correlation of detection frequencies between the CFS and HC samples for the remaining 98 proteins (R^2 ^= 0.70).

Because of the congruent proteomes from the pooled and individual samples, data from cohorts 1 and 2 were combined for further analyses (n = 24 total samples). A total of 115 unique proteins were detected (table 2). HC had 72 proteins and CFS 83. These groups shared 40 proteins. A set of 44 proteins were detected at low frequency in only 1 or 2 subjects from one group or the other. The frequencies of detection of shared proteins were highly correlated between the 2 groups of subjects (figure 5). The slope of the regression line was 0.93, the intercept 0.05, and R^2 ^= 0.70.

### CFS – related proteome from all samples

Nineteen proteins were detected significantly more frequently in CFS than HC samples (p < 0.05 by ANOVA) (table 3). Chance would predict that as many as 1 out of 20 proteins may have been shared by the two proteomes (p = 0.05). However, detection of 19/115 (0.165) suggested that nonrandom pathological processes were active in generating the CFS proteome. The 19 CFS – related proteins were shifted upwards from the line of identity shown on figure 5. The proteins were detected in 27% to 64% of CFS, and 0% to 23% of HC samples. Fisher's Exact test was significant for 16 of these proteins (table 3).

Nine proteins were found consistently in cohorts 1 and 2 and the combined sample (table 3). Plasma proteins included α-2-mac, ceruloplasmin, ORM2, and C4A/C4B. Brain – derived proteins were APLP1, autotaxin, PEDF and the structural proteins BEHAB and gelsolin. Cohort 2 and the total sample showed significant differences between CFS and HC for keratin proteins K6C, K16 and K17. Conversely, cohort 1 and the combined group both detected C3, α-1-antichymotrypsin, and haptoglobin significantly more frequently in CFS than HC. The combined group of 24 samples produced 4 proteins that were not significantly different between CFS and HC in the smaller cohorts 1 and 2. Hemopexin was probably from plasma. Ig λ was likely a marker of plasma immunoglobulin influx, although local B lymphocyte synthesis was possible. Significant brain – derived polypeptides were chromogranin B and secreted phosphoprotein 1 (osteopontin). Seven proteins were significantly different by ANOVA but not Fisher's Exact test. The larger overall group size (n = 24) was responsible for the differences in statistical significance compared to cohort 2 (n = 21).

Fourteen of the proteins were not detected in HC samples (0% detection) (table 3). They may have been present in HC at levels below the lower limit of detection of the mass spectrometer. Future qualitative studies starting with larger amounts of cerebrospinal fluid protein may identify these proteins in HC subjects. Quantitative studies will be required to determine if concentrations were different between CFS and HC. These could not be performed here because of the insufficient volumes of cerebrospinal fluid that remained. These findings suggest that additional samples may have increased the size of the CFS – related proteome detected by MS-MS.

### Shared cerebrospinal fluid proteome from all samples

The 16 most prevalent proteins (frequencies of detection ≥ 50%) had both plasma and central nervous system origins ' [see Additional file 1]'. Albumin, Ig γ1, Ig γ4, transferrin, α-1-antitrypsin and the apolipoproteins E (including isotypes 3 and 4), J, and A-I were probably of plasma origin. Prostaglandin D2 synthase, transthyretin, and angiotensinogen were of brain origin. Dickkopf related protein-3 likely inhibited neural growth in these adult brains. The precise targets of leptomeningeal cystatin C were unclear, but its high frequency of detection highlighted the importance of secreted cysteine protease inhibitors in normal central nervous system function. Epithelial cells of the choroid plexus or arachnoid membranes were the probable sources of the keratins. Epidermal contamination with keratins 1, 9 and 10 during the lumbar punctures or processing could not be ruled out ' [see Additional file 1]'.

Plasma proteins detected with frequencies of 25% to 49% included hemoglobins, immunoglobulin chains, and orosomucoid 1 ' [see Additional file 1]'. Structural proteins (tubulins, keratins, actin β, and secreted phosphoprotein 1) were common. The latter was associated with CFS. Regulatory proteins included chromogranin A and the neuron directional marker disco-interacting protein 2 [*Drosophila*] (DIP2). Many of the proteins had multiple domains and may have had additional functions *in vivo*.

**Figure 6:**
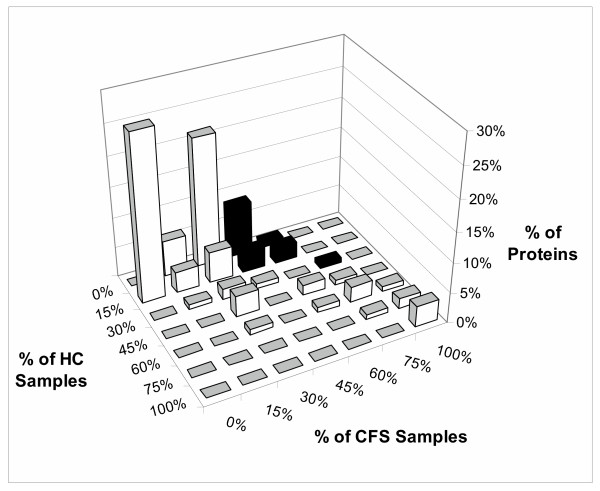
Distributions of proteins in healthy control (HC) and CFS samples. The frequency of detection for each protein was determined for the HC (left axis) and CFS (right axis) groups. These axes were divided into "bins" of 0% (absent), 1 to 15%, 16 to 30%, 31 to 45%, 46 to 60%, 61 to 75%, and 76 to 100%. The vertical axis was the percentage of all proteins detected within each intersection of the CFS vs. HC matrix. Most of the proteins were detected in less than 30% of each group. Proteins detected in both groups with roughly equal frequencies of detection were near the line of identity (white bars). The grid region corresponding to the CFS – associated proteome was highlighted by black bars.

A large number of proteins were detected in 3 or fewer samples ' [see Additional file 1]'. This was apparent by converting figure 5 into a 3 – dimensional plot showing the numbers of proteins detected in the HC and CFS groups (figure 6). Discrete ranges of frequencies ("bins") were selected. The seven bins were 0, increments of 15% up to 75%, and the upper 25%. The bins generated a grid that was analogous to figure 5. The vertical axis showed the percentages of total proteins that were present at each intersection point on the grid. Most of the proteins were grouped in the low frequency intersection points (<30% detection in both groups). A small number of cerebrospinal fluid proteins with known high concentration were detected in most samples. These shared proteins extended the line of identity to near the 100% detection point. The high concentrations of this small set of proteins provided the rationale for removing these proteins in order to assess lower abundance proteins. The CFS – associated proteome was identified along the 0%, 1 to 15%, and 16 to 30% HC grids, and the 31% to 75% grids of the CFS group (figure 6).

### Novel proteins from all samples

Overall, 62 of the 115 proteins had not been detected in previous studies of cerebrospinal fluid ' [see Additional file 1]'. These included DIP2, neuronal PAS domain protein 2 (seasonal affective disorder – related), additional sex combs – like protein 1 (ASXL1), and neuroglobin. Several were represented by only a single, highly selective peptide. Keratins 5, 6c, 6e, 14, 16, and 17 were the largest single protein family to be newly described.

### Depiction as a two-dimensional electrophoresis gel

**Figure 7:**
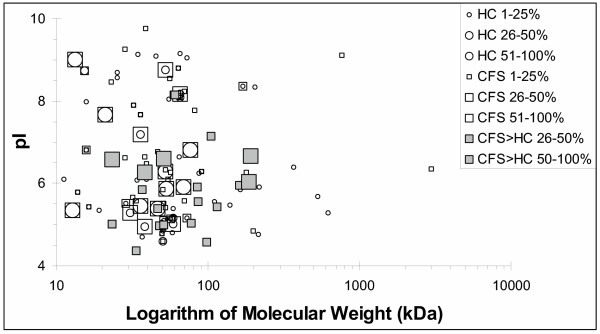
Isoelectric point (pI) vs. logarithm of molecular weight. The frequencies of detection for proteins in the healthy control (HC) group were graded as 1 to 25% (small circles), 26 to 50% and 51 to 100% (large circles). Proteins detected in the CFS group (open squares) were similarly graded. The CFS – associated proteins detected in 26 to 50% and 51 to 100% of samples were depicted as smaller and larger grey squares, respectively.

The proteins from the HC and CFS groups were graphed based on predicted molecular weights and isoelectric points (figure 7) ' [see Additional file 1]'. The cerebrospinal fluid proteins, including the CFS – associated proteins, were most concentrated between isoelectric points of 4.7 and 7, and molecular weights of 30 to 110 kDa. The shared proteins appeared as coincident circles (HC) within squares (CFS). The proteins significantly associated with CFS (p < 0.05 compared to HC for frequency of detection) had molecular weights between 25 kDa and 110 kDa. They fell into 2 groups. Five proteins were detected in over half of CFS samples, and had pI's between 6 and 7. Twelve CFS – related proteins were detected in 26% to 50% of CFS samples and had pI's between about 4.5 and 6.

## Discussion

The discovery of shared cerebrospinal fluid proteins between two independent cohorts of CFS subjects that were not present in two independent sets of healthy control subjects demonstrated that there was differential protein expression in this syndrome (table 3). There was significant overlap of CFS, PGI and FM syndromes within the CFS group (figure 1). The mix of co-morbid syndromes was different for cohorts 1 and 2, yet the proteomic studies identified a single CFS – related proteome.

Both the individual proteins and the patterns of proteins may offer insights into CFS pathophysiology, and serve as potential biomarkers for CFS diagnosis and the assessment of disease severity. The variable ***B***_***1/5 ***_epitomized the qualitative pattern recognition of this proteomic "biosignature". The statistical model was predictive of CFS status regardless of which of the 5 proteins, or how many of them, were detected. Prospective studies will be required to determine the sensitivity, and specificity of the proteomic pattern and optimal logistic model(s) for discriminating CFS and related syndromes from normal, idiopathic fatigue, illness-related fatigue states, affective disorder, chronic pain states such as fibromyalgia and regional pain syndromes, and inflammatory central nervous system disorders [1-7]. These studies of larger numbers of subjects would be anticipated to refine the CFS – related proteome and the terms of the biosignature variable. These prospective studies are also required to test the objective nature of biosignature variable and other CFS – related proteins for predicting CFS status.

The basis for differences between CFS and HC was the ability of the mass spectrometer to detect certain proteins in CFS but not HC. This concept of "detectability" is critical to understanding the qualitative nature of our results and predictive model. Improvements in the lower limits of detection may identify a larger number of low abundance proteins. These may include the cerebrospinal fluid proteins that were relatively more abundant (and detectable) in CFS compared to HC subjects. This remains to be tested. Quantitative studies will be required to confirm the qualitative distinctions between the CFS and HC groups.

The origins and putative functions of the CFS – related proteome may provide clues to CFS pathophysiology. Cerebrospinal fluid is generated by (a) selective diffusion and transport of plasma components through the choroid plexus and brain parenchymal vessels, (b) synthesis by the choroid plexus and meningeal epithelia, (c) secretion from brain neuron, glia, and other parenchymal cells into the local interstitial fluid, and (d) release from injured and apoptotic parenchymal, mucosal and leukocytic cells [43]. Plasma protein flux is regulated by transport through the blood-brain barrier and the efflux of protein from cerebrospinal fluid through arachnoid granulations into venous blood [43,44]. Alternatively, the fluid may exit the meningeal space through perinasal and olfactory lymphatics [45]. Dysfunction of hydrodynamic plasma influx or cerebrospinal fluid efflux may contribute to the variations in relative detectability of brain versus plasma proteins in the CFS proteome [46]. Decreased plasma influx would lead to relatively higher abundances of proteins synthesized in the brain. These proteins may have become easier to detect in CFS. Brain parenchymal mast cells may regulate brain microvascular permeability, possibly through histamine release [47]. If so, this could explain the benefits of tricyclic drugs such as doxepin and imipramine that have potent histamine receptor 1 antagonist activities [48]. Histamine receptor 1 antagonists that do not cross the blood brain barrier have no benefit in CFS [49].

Proteins of plasma, choroid plexus, or meningeal origin included α-2-mac [50], orosomucoid 1 and 2 [51], α1-antichymotrypsin [52], complement factor 4B precursor [53], and ceruloplasmin [54]. These antiproteases, antioxidant, pro- and anti-inflammatory proteins suggested activation of the cerebrospinal innate immune system. Secreted central nervous system proteins included PEDF [55], CNDP1 [54], and autotaxin [56].

ORM2 is a glycosylated plasma lipocalin with a hydrophobic pocket that binds a wide variety of drugs, hemin, progesterone and the CCR5 receptor on macrophage – lineage cells [57,58]. Both ORM2 and ORM1 are acute phase reactants that are synthesized in the liver, but may also be synthesized at sites of brain injury or astroglial cell activation [59]. Like haptoglobin [60] and hemopexin [61], ORM2 and ORM1 may contribute to heme and iron sequestration in the central nervous system in CFS. Iron sequestration is an important antioxidant and antibacterial innate immune defense function [62]. Haptoglobin and apolipoprotein J act as extracellular chaperone proteins *in vivo *[63]. They may exert anti-inflammatory actions by inhibiting the inappropriate self-association of "damaged" (misfolded) extracellular proteins.

The presence of heme sequestering proteins begged the question of whether free hemoglobin was present in cerebrospinal fluid. This was the case since hemoglobins α1 and α2, β, β Sickle, and δ were detected in the cerebrospinal fluid proteome shared by all subjects ' [see Additional file 1]'. The source of hemoglobin in normal cerebrospinal fluid could have been the lumbar puncture. If so, the detection rate for hemoglobin would be expected to be similar for all groups, as was found. However, this did not explain the significantly more frequent detection of heme scavengers in CFS (55%) compared to HC (15%) samples (table 3). Apolipoprotein B has been used as a marker for the acute introduction of plasma into the cerebrospinal fluid since this protein is not synthesized in the brain [64]. Apolipoprotein B was detected only once (CFS) ' [see Additional file 1]', indicating that the lumbar punctures were not a consistent cause of hemorrhage [64]. Free hemoglobin levels (and mass spectrometric detection) may be related to haptoglobin isoforms [65]. We have not evaluated this potential correlation in our population.

A number of central nervous system conditions may lead to localized bleeding with hemoglobin release with the induction of heme sequestering proteins. One large group meeting these characteristics are the cerebral amyloid angiopathies (CAA) (cerebrovascular amyloidosis) [66]. CAA syndromes are defined by protein misfolding, perivascular amyloid deposition, weakening of vessels walls, microhemorrhages to severe cerebral infarction, and dementia or sudden death occurring in the 3^rd ^to 5^th ^decades. The CFS spectrum of illnesses do not demonstrate higher than normal rates of these causes of death making it unlikely that any of the currently identified CAA syndromes were responsible. However, we hypothesize that a mild, focally transient or reversible form that does not lead to either permanently damaging or lethal hemorrhage or dementia may occur in CFS.

This hypothesis would explain many of the parallels between the proteins associated with CAA syndromes and the CFS – related proteome. Gelsolin is an actin "capping" protein that terminates actin polymerization [67]. Gelsolin cleaved by capsase-3 leads to the "blebbing" during apoptosis. Mutant gelsolin isoforms lead to misfolding and the perivascular amyloid fibril deposition in Finnish type cerebrovascular amyloidosis [66,68]. Gelsolin amino acid sequences 173–243 and 173–202 are α-helices that are converted to β – pleated sheet conformations in amyloidosis [68]. The proteases responsible for the gelsolin cleavage that permit the change in secondary structure are unknown. An E^693^Q mutation in amyloid β (A4) precursor-like protein 1 (APLP1) leads to hereditary Dutch type – cerebral hemorrhage with amyloidosis ' [see Additional file 1]' [69]. This is separate from the involvement of this protein in Alzheimer's disease. Immunoglobulin lambda (Ig λ) light chains are relatively unstable, have a tendency to unfold and polymerize [70], and have been associated with APLP1 in an intracerebral syndrome [71], and systemic amyloid syndromes with and without B lymphocyte dyscrasias [72].

Cystatin C, a leptomeningeal inhibitor of papain – like cysteine proteases, was found in HC and CFS samples. Cystatin C is a homodimer with several domains. The random coil polypeptide linking the terminal domain to the rest of the protein can be proteolytically cleaved [73]. The "free" domains refold their tertiary structure from constrained α – helices to β – pleated sheets with lower free energies [74]. These domains are then reattached to the opposite member of the homodimer. This "protein swapping" mechanism is analogous to a DNA recombination – like process [73]. Inactive β – pleated sheet domains may then polymerize into amyloid deposits. Autosomal dominant Icelandic CAA is due to the disease-causing L68Q variant of human cystatin C [75]. Cystatin C amyloid immunoreactive material has been found in cerebral cortical, white matter parenchymal and leptomeningeal vessels [74]. Deposition was more prominent in the media of parenchymal vessels and in the adventitia of leptomeningeal vessels. Complexes of cystatin C or Ig λ with APLP1 have been found in extracellular deposits.

Transthyretin, a thyroxine transporting member of the albumin family, was a common brain – derived component of cerebrospinal fluid that was not part of the CFS proteome ' [see Additional file 1]'. Misfolding of transthyretin (meningovascular amyloidosis), angiotensinogen, β2 – microglobulin, lysozyme, the Notch3 gene product, and the familial prion protein may each lead to amyloidosis [66,74,76-78]. Acquired prion diseases may potentially contribute to the CFS – spectrum of illnesses, but the predilection for females and other epidiomological findings make this an unlikely pathological event.

Other components of the CFS – related proteome promote amyloid deposition. Complement factors C3, C4 and B become activated in amyloidosis and Alzheimer's disease [79]. Apolipoproteins E, E4, and J, and microtubule-associated protein 2 have been associated with CAA syndromes and Alzheimer's disease [66,80]. Apolipoprotein E4 may target the amyloid to vessel walls. Chromogranin B – immunoreactive material (table 3) was found in 15% of plaques in Alzheimer's disease [81]. There was a significant loss of chromogranin B – immunoreactivity in the dorsolateral, the entorhinal, and orbitofrontal cortex in Alzheimer's disease. The absence of chromogranin B in these anatomical locations could result in defective synaptic function and the loss of neurohormonal effects. Chromogranin B – immunoreactive material was selectively associated with prion protein deposits in Creutzfeldt-Jakob disease. In contrast, chromogranin A was seen only in amyloid β plaques of Alzheimer's disease [82].

Chromogranin B is a highly multifunctional protein. It is a high capacity, low affinity calcium (Ca^2+^) storage protein that complexes to the inositol 1,4,5-trisphosphate receptor (InsP3R) in the endoplasmic reticulum. Thus, chromogranin B may modulate Ca^2+ ^release [83]. Chromogranin B (CGB) is a prohormone that can be cleaved to release secretogranin I precursor (Sg1), GAWK and CCB peptides. Both chromogranins B and A are prohormones for the antimicrobial peptides vasostatin-1 and secretolytin [84]. mRNA for chromogranin B was detected in human monocytes, and may be present in other macrophage/monocytic lineages such as astroglial cells in the brain.

Pigment epithelium-derived factor (PEDF) (table 3) is another multipurpose CNS protein [55,85]. Although a member of the serpin (serine antiprotease) protein family, it does not possess this activity. PEDF has antiangiogenic activity that may prevent or reduce neovascularization after retinal or cerebral hemorrhage. Both PEDF and a 44 amino acid long proteolytic fragment have potent anti-vascular permeability effects [86]. PEDF controls the transit of neurons through the cell cycle, promoting their entry into a quiescent state [87]. The protein may protect neurons and potentially glial cells from apoptosis. Protein levels in the eye and brain decrease with age, but this cannot explain the increase in detection rate in CFS since age was not significantly different between groups. Dickkopf-3, another protein that limits neural proliferation [88], was detected in 46% of samples.

Autotaxin (TEFLSNYLTNVDDITLVPGTLGR) is a 23 amino acid peptide cleaved from the middle of ectonucleotide pyrophosphatase/phosphodiesterase 2 (ENPP2 gene) [56,89,90]. The parent protein possesses both nucleotide pyrophosphatase and lysophospholipase D (lyso-LPD) activities [56,91]. Aliases include phosphodiesterase-1α, phosphodiesterase I/nucleotide pyrophosphatase 2, and alkaline phosphodiesterase I. This "tumor mobility peptide" enhances metastasis of breast and other cancer [91]. Its unclear if the pyrophosphatase, lysophospholipase D, or autotaxin functions are more important in the brain and in CFS.

Chromogranin B, PEDF, autotaxin, angiotensinogen, and other polypeptides are significant prohormones. The protease cascades that lead to their cleavage and the release of active neuropeptides are poorly understood. However, the peptide hormone effects must be potent since plasma and brain – derived protease inhibitors were detected more frequently in CMI than in HC (table 4).

α2-Macroglobulin [92] and α-1-antichymotrypsin were detected in the CFS proteome (table 3). IL-1 may induce a mutant promoter allele of α-1-antichymotrypsin that leads to increased central nervous system and the promotion of Alzheimer disease [93]. Angiotensinogen also has serine protease inhibitor properties. Angiotensinogen was detected in about half of all samples and was not associated with CFS. Haplotype B may be related to microangiopathy – related cerebral damage (MARCD) that can lead to cognitive impairment and gait disturbances in the elderly [78]. This protein is synthesized by glial cells [94,95]. The C-terminal has serine protease inhibitor activity that inhibits angiogenesis. Angiotensins I, II and III are cleaved from the N-terminal. Angiotensin II and III may bind to angiotensin 4 receptors (also known as insulin-regulated aminopeptidase [94]) in hypothalamic and brainstem nuclei to stimulate the sympathetic nervous system (increase systemic blood pressure), sodium and thermal regulation. The remaining 97% (des [Ang I]angiotensinogen) has no assigned function.

The presence of keratin 16 suggests dysfunction in the leptomeningeal and choroid plexus epithelial system in CFS. Keratin expression in the central nervous system has been incompletely studied, with most of the focus placed on neoplastic tissues [96-98]. Keratin 16 is upregulated in epidermal diseases such as psoriasis [99]. Epidermal growth factor, interferon-γ and *ras *can stimulate *Sp1 *and *jun *(AP1) proteins that activate the keratin 16 promoter. By analogy, we hypothesize that the presence of keratin 16 in the CFS – associated proteome was an indication of epithelial cell activation within the central nervous system in CFS.

BEHAB may play a role in central nervous system repair or remodeling processes [100]. Its 2 isoforms bind to extracellular matrix hyaluronan. The full length isoform is secreted into the extracellular matrix. The shorter, splice variant may be linked to glycophosphatidylinositol and form a cell surface protein. The longer variant is highly expressed in childhood, then reaches low, steady state cerebrospinal fluid concentrations by age 20. The shorter variant maintains uniformly low levels throughout development. BEHAB mRNA is elevated 7-fold in gliomas suggesting that glial cells are the normal source *in vivo*. BEHAB is also increased in response to brain injury. Glial or other cells with BEHAB anchored to their external plasma membranes may be attracted to putative areas of tissue injury where extracellular matrix became exposed, or where hyaluronan was secreted.

An alternative to the CAA hypothesis is glial cell activation [101] with the release of innate immune and regulatory factors. Activation of leptomeningeal cells with the secretion of several of the proteins listed above is also possible. CFS syndromes may be initiated by unknown factors that activate these cells, or they may activate anti-inflammatory and innate immune defenses as a result of the original insult. These possibilities may be addressed in future studies and by comparison of the proteomes from CFS subjects with different durations or patterns of illness.

Several novel proteins were identified. These included Dickkopf-3 [88], disco-interacting protein 2 [*Drosophila*] (DIP2) [102], neuronal PAS domain protein 2 (seasonal affective disorder – related) [103], additional sex combs – like protein 1 (ASXL1) [104], and neuroglobin [23]. Several were represented by only a single, highly selective peptide ' [see Additional file 1]'. Keratins 5, 6c, 6e, 14, 16, and 17 were the largest single protein family to be newly described [96-99].

No proteins were significantly associated with the good health of the HC subjects.

## Conclusion

This pilot investigation demonstrated that the CFS, PGI and FM subjects had a significant overlap between their syndromes. Despite the differences in their original case designations, they had very similar responses on questionnaire, quality of life and nociceptive measures. Again, despite the differences in the diagnostic label applied to them for study entry, their cerebrospinal fluid proteomes demonstrated reproducible constituents. The CFS – related proteome was essentially the same for the two independent CFS cohorts. The proteome was remarkable for the number of proteins associated with protein misfolding and cerebrovascular amyloidosis syndromes. These included gelsolin, amyloid β protein (APLP1), Ig λ, C3, C4, chromogranin B, α2-macroglobulin and α-1-antichymotrypsin antiproteases, the heme and iron scavengers haptoglobin, hemopexin, and orosomucoid 2, angiogenic and antiangiogenic prohormones such as autotaxin and PEDF, and the structural proteins gelsolin, BEHAB and keratin 16. Their presence in the CFS – associated proteome suggested a potential pathophysiological link. We propose the hypothesis that CFS may be a nonlethal, protein – misfolding, cerebrovascular amyloidosis – like syndrome.

An objective statistical model was derived to predict CFS status based only on the proteomic detection of keratin 16, α-2-macroglobulin, orosomucoid 2, autotaxin, and pigment epithelium-derived factor. This is the first predictive model of CFS to be based only on objective data. This legitimizes our hypothesis that a common central nervous system pathophysiology was present in the CFS spectrum of illnesses. Despite the many combinations of "labels" (e.g. CFS, FM, PGI) applied to each subject (figure 1), only one, consistent CFS – related proteome was obtained from the two independent sets of subjects (table 3). Individual proteins or their patterns of detection ("biosignatures" [14,18-20]) may prove to be valuable biomarkers in diagnostic assays. These assays may gauge disease severity, dynamic variations in symptomatology, and longitudinal alterations with age or treatments. Given the continued controversy over whether CFS and its allied syndromes are legitimate medical conditions, our proteomic model provides initial objective evidence for the legitimacy of CFS as a distinct neurological disease.

## Abbreviations

aa, amino acids; α-2-mac, α-2-macroglobulin; APLP-1, amyloid precursor like protein 1; BEHAB, **b**rain **e**nriched **h**y**a**luronan **b**inding **p**rotein; chondroitin sulfate proteoglycan, brevican; CAA, cerebral amyloid angiopathy; CapLC-QToF, capillary liquid chromatography – quadrupole – time of flight; CFS, chronic fatigue syndrome; CMI, chronic multisymptom illness; CRH, corticotrophin releasing hormone; GLM, general linear model; K16, keratin 16; MS, mass spectrometry; MS-MS, tandem mass spectrometry; ORM2, orosomucoid 2; ***B***_***1/5***_, a biosignature variable representing the presence of any 1 of the 5 CFS-related proteins in the multiple logistic model for predicting CFS status; PEDF, pigment epithelium derived factor; PGI, Persian Gulf War Illness; PIR, Protein Information Resource.

## Competing interests

The author(s) declare that they have no competing interest.

## Authors' contributions

The original study was organized by DJC. BC performed the proteomic analysis with the assistance of LKP and SH. BC and JNB performed the bioinformatics that were modeled by HM. The manuscript was written by JNB.

## Pre-publication history

The pre-publication history for this paper can be accessed here:



## Supplementary Material

Additional File 1 The frequencies of detection for cerebrospinal fluid proteins identified by mass spectrometry in healthy control and chronic fatigue syndrome specimens. Click here for file
