# Supplementary material for: A chronic fatigue syndrome – related proteome in human cerebrospinal fluid
Source: BMC Neurol. 2005 Dec 1;5:22. doi: 10.1186/1471-2377-5-22 (PMC1326206; doi:10.1186/1471-2377-5-22)
Supplement: Additional File 1 — The frequencies of detection for cerebrospinal fluid proteins identified by mass spectrometry in healthy control and chronic fatigue syndrome specimens. [file 1471-2377-5-22-S1.doc]

‘[Additional file 1]’. The frequencies of detection for cerebrospinal fluid proteins identified by mass spectrometry in healthy control (HC; n=13) and chronic fatigue syndrome (CFS; n=11) specimens. Proteins detected more frequently in CFS than HC were identified by ANOVA (*, CFS column, from table 3). Proteins detected in cerebrospinal fluid for the first time (n = 62) were indicated (**^§^**). NCBI GeneID, locus or accession number, gene name abbreviation, molecular weight (MW, kDa), isoelectric point (pI), number of peptides identified by mass spectrometry (Pept #) and percent coverage (% Cover) were given for each protein.

| GeneID | Locus or Accession | Proteins, Aliases, and Gene Name Abbreviations | HC  n=13 | CFS  n=11 | MW (kDa) | pI | Pept  # | % Cover |
| --- | --- | --- | --- | --- | --- | --- | --- | --- |
| 171023 | Q8IXJ9 | Additional sex combs-like protein 1; ASXL1 ^§^ | 0.15 | 0 | 165.4 | 5.85 | 1 | 1% |
| 213 | P02768 | Albumin; ALB | 1 | 0.91 | 69.2 | 5.91 | 77 | 58% |
| 351 | M15533 | Amyloid precursor-like protein 1, APLP1 (β, A4); protease nexin-II, Alzheimer disease; APP | 0 | 0.36 * | 86.9 | 5.54 | 5 | 6% |
| 183 | P01019 | Angiotensinogen; AGT | 0.62 | 0.64 | 53.2 | 5.87 | 12 | 24% |
| 301 | P04083 | Annexin A1; lipocortin I, calpactin II, chromobindin 9, phospholipase A2 inhibitory protein; ANXA1 ^§^ | 0 | 0.09 | 38.6 | 6.64 | 1 | 3% |
| 462 | P01008 | Antithrombin III; SERPINC1 | 0 | 0.18 | 52.6 | 6.32 | 4 | 17% |
| 335 | P02647 | Apolipoprotein A-I; APOA1 | 0.38 | 0.64 | 30.8 | 5.28 | 11 | 33% |
| 338 | P04114 | Apolipoprotein B100 (apolipoprotein B-48); APOB ^§^ | 0 | 0.09 | 28.6 | 6.61 | 4 | 15% |
| 348 | P02649 | Apolipoprotein E; APOE | 0.85 | 0.82 | 36.1 | 5.45 | 11 | 31% |
| 1191 | P10909 | Apolipoprotein J; clusterin; complement lysis inhibitor, CLI; SP440; sulfated glycoprotein-2; testosterone-repressed prostate message 2; clusterin; CLU | 0.62 | 0.82 | 52.5 | 6.27 | 12 | 25% |
| 5168 | NP_006200.2 | Autotaxin; ectonucleotide pyrophosphatase; phosphodiesterase Iα; phosphodiesterase 2; alkaline phosphodiesterase I; plasma lysophospholipase D; ENPP 2 | 0 | 0.36 * | 105.2 | 7.14 | 5 | 5% |
| 63827 | AAG23135 | Brain enriched hyaluronan binding, BEHAB; brain chondroitin sulfate proteoglycan; brevican; BCAN | 0 | 0.36 * | 99.1 | 4.57 | 5 | 6% |
| 696 | Q13410 | Butyrophilin subfamily 1 member A1; BTN1A1 ^§^ | 0.08 | 0 | 59 | 5.38 | 1 | 2% |
| 55195 | NP_060638 | C14orf105 ^§^ | 0.08 | 0 | 34.6 | 9.13 | 1 | 4% |
| 84735 | NP_116038 | Carnosine dipeptidase 1, CNDP1; carnosinase 1, CN1; glutamate carboxypeptidase-like II, metallopeptidase M20.006 | 0.08 | 0.36 | 56.7 | 5.12 | 4 | 8% |
| 56996 | AAK21009 | Cation-chloride cotransporter-interacting protein 1 isoform b; SLC12A9 ^§^ | 0.08 | 0 | 66 | 9.14 | 1 | 2% |
| 57514 | NP_065805 | CDc42 GTPase-activating protein; CDGAP ^§^ | 0 | 0.18 | 50.4 | 5.85 | 6 | 13% |
| 13566 | P00450 | Ceruloplasmin (EC 1.16.3.1); ferroxidase; CP | 0 | 0.36 * | 115 | 5.43 | 6 | 5% |
| 1113 | P10645 | Chromogranin A (CgA); pituitary secretory protein 1; parathyroid secretory protein 1; contains vasostatin-1, vasostatin-2, EA-92, ES-43, pancreastatin, SS-18, WA-8, WE-14, LF-19, AL-11, GV-19, GR-44, ER-37; CHGA | 0.31 | 0.18 | 50.7 | 4.58 | 5 | 11% |
| 1114 | P05060 | Chromogranin B (CgB); secretogranin 1; contains GAWK and CCB peptides | 0 | 0.36 * | 78.3 | 5.02 | 3 | 4% |
| 710 | NP_000053 | Complement C1 esterase inhibitor precursor; SERPING1 | 0 | 0.18 * | 55.1 | 6.09 | 3 | 11% |
| 718 | P01024 | Complement C3 (contains C3a anaphylatoxin) | 0.23 | 0.64 * | 187.2 | 6.02 | 29 | 17% |
| 720, 721 | CAI41750 | Complement C4A (gi|57209522) and C4B (gi|38649251) gene products | 0 | 0.55 * | 192.3 | 6.66 | 16 | 13% |
| 1539 | NP_001331 | Cylicin 2; sperm head structural protein; CYLC2 ^§^ | 0 | 0.18 | 39 | 9.74 | 2 | 6% |
| 1471 | NP_000090 | Cystatin C precursor; neuroendocrine basic polypeptide, γ-trace, post-γ-globulin protein; CST3 | 0.54 | 0.55 | 13.3 | 9 | 9 | 74% |
| 117159 | NP_444513 | Dermcidin precursor; AIDD protein; proteolysis inducing factor of sweat gland; DCD | 0.08 | 0 | 11.3 | 6.09 | 1 | 9% |
| 27122 | Q9UBP4 | Dickkopf related protein-3 precursor; DKK3 | 0.38 | 0.55 | 38.3 | 4.95 | 5 | 14% |
| 23181 | NP_055966 | Disco-interacting protein 2 [*Drosophila*]; DIP2-like isoform a; putative polycomb group protein; C21orf106 ^§^ | 0.23 | 0.27 | 170.4 | 8.35 | 5 | 3% |
| 1787 | AAK68033.1 | DNA (cytosine-5-)-methyltransferase 2; DNMT2 ^§^ | 0.08 | 0 | 9.7 | 9.57 | 1 | 9% |
| 5436 | NP_002687 | DNA-directed RNA polymerase II polypeptideG;POLR2G^§^ | 0.08 | 0 | 19.3 | 5.33 | 1 | 7% |
| 667 | Q03001 | Dystonin isoform 1; bullous pemphigoid antigen 1 isoforms 1/2/3/4/5/8, 230 kDa bullous pemphigoid antigen, BPA, hemidesmosomal plaque protein, dystonia musculorum protein; DST ^§^ | 0.08 | 0 | 372.2 | 6.38 | 1 | 1% |
| 25975 | NP_056322 | EGF-like-domain, multiple 6; epidermal growth factor-like protein 6; EGFL6 ^§^ | 0.08 | 0 | 78.5 | 5.03 | 1 | 1% |
| 2243 | P02671 | Fibrinogen α; FGA | 0 | 0.18 | 69.8 | 8.23 | 1 | 2% |
| 2244 | P02675 | Fibrinogen β (contains fibrinopeptide B); FGB | 0 | 0.18 | 55.9 | 8.54 | 1 | 10% |
| 2934 | CAI14415 | Gelsolin, amyloidosis, Finnish type; GSN | 0 | 0.45 * | 85.7 | 5.9 | 7 | 33% |
| 2638 | P02774 | Group-specific component; vitamin D binding protein (lipocalin); GC | 0 | 0.18 | 53 | 5.4 | 2 | 4% |
| 3977 | NP_000846 | Guanylate cyclase 1, soluble, α 2; GUCY1A2 ^§^ | 0 | 0.09 | 81.7 | 7.77 | 1 | 1% |
| 3240 | P00738 | Haptoglobin; HP | 0.15 | 0.55 * | 38.5 | 6.26 | 7 | 20% |
| - | U56725.1 | Heat shock protein 70, 2, HSP70.2 ^§^ | 0 | 0.18 | 70 | 5.56 | 1 | 2% |
| 8925 | NP_003913 | Hect; homologous to the E6-AP (UBE3A) carboxyl terminus domain and RCC1 (CHC1)-like domain (RLD) 1; guanine nucleotide exchange factor p532; HERC1 ^§^ | 0.08 | 0 | 532.2 | 5.68 | 1 | 1% |
| 3039,  3040 | P69905 | Hemoglobin α 1, 2; HBA1, HBA2 | 0.38 | 0.27 | 15.3 | 8.72 | 18 | 50% |
| 3043 | P68871 | Hemoglobin β, HBB | 0.15 | 0.36 | 15.9 | 6.8 | 7 | 48% |
| 3043 | P68871 | Hemoglobin β Sickle ^§^ | 0 | 0.18 | 15.9 | 6.8 | 6 | 52% |
| 3045 | P02042 | Hemoglobin δ; HBD ^§^ | 0.08 | 0 | 15.9 | 7.97 | 1 | 7% |
| 3263 | P02790 | Hemopexin; β-1-B-glycoprotein; HPX | 0.15 | 0.55 * | 51.3 | 6.59 | 14 | 30% |
| 22944 | NP_036443 | HsKin 17 protein; HAS kin17 protein; recA; KIN ^§^ | 0.15 | 0 | 45.4 | 9.07 | 1 | 2% |
| 84074 | NM_032134 | Hypothetical protein DKFZp434P0316; LOC84074 ^§^ | 0 | 0.18 | 180.8 | 6.25 | 2 | 1% |
| 57476 | XP_370660 | Hypothetical protein KIAA1201 protein (GRAM) ^§^ | 0.08 | 0 | 88.1 | 6.23 | 1 | 1% |
| 3493 | P01876 | Ig α1; IGHA1 | 0.08 | 0.27 | 73.4 | 5.14 | 8 | 12% |
| 3500 | P01857 | Ig γ1; IGHG1 | 0.46 | 0.73 | 52.7 | 8.74 | 67 | 50% |
| 3501 | P01859 | Ig γ2; IGHG2 | 0.23 | 0.18 | 35.9 | 7.66 | 5 | 15% |
| 3502 | X99549.1 | Ig γ3; IGHG3 | 0.08 | 0.09 | 32.3 | 7.89 | 2 | 4% |
| 3503 | P01861 | Ig γ4; IGHG4 | 0.46 | 0.73 | 35.9 | 7.18 | 6 | 14% |
| 50802 | S40354 | Ig κ; IGK@ | 0.23 | 0.36 | 28.8 | 5.5 | 10 | 37% |
| 3535 | LVHU2 | Ig λ: IGL@ | 0.15 | 0.64 * | 23.4 | 6.58 | 6 | 27% |
| 3419 | NP_005521 | Isocitrate dehydrogenase 3 (NAD+) α; IDH3A ^§^ | 0 | 0.18 | 39.6 | 6.46 | 2 | 6% |
| 3848 | O76013 | Keratin 1, KRT1 | 0.46 | 0.73 | 65.5 | 8.16 | 38 | 44% |
| 3849 | CAF31523 | Keratin 2a, KRT2A ^§^ | 0.39 | 0.27 | 65.5 | 8.07 | 17 | 28% |
| 3851 | NP_149034 | Keratin 4; KRT4 ^§^ | 0 | 0.09 | 57.3 | 6.25 | 4 | 7% |
| 3852 | P13647 | Keratin 5; KRT5 ^§^ | 0.08 | 0.27 | 62.4 | 8.14 | 13 | 18% |
| 3853 | NP_005545 | Keratin 6a; KRT6A ^§^ | 0 | 0.09 | 59.9 | 8.14 | 5 | 9% |
| 3854 | P04259 | Keratin 6b; KRT6B ^§^ | 0 | 0.09 | 59.9 | 8.14 | 14 | 17% |
| 140446 | P48666 | Keratin 6c; KRT6C ^§^ | 0 | 0.27 * | 60.8 | 8.14 | 21 | 31% |
| 286887 | P48668 | Keratin 6e; KRT6E ^§^ | 0.08 | 0.27 | 59.8 | 8.14 | 6 | 11% |
| 3855 | NP_005547.3 | Keratin 7; sarcolectin; KRT7 ^§^ | 0 | 0.18 | 51.3 | 5.5 | 3 | 6% |
| 3857 | P35527 | Keratin 9; KRT9 | 0.46 | 0.18 | 59.4 | 5.14 | 18 | 33% |
| 3858 | P13645 | Keratin 10; KRT10 | 0.31 | 0.55 | 59.5 | 5.01 | 13 | 24% |
| 3861 | NP_000517 | Keratin 14; KRT14 ^§^ | 0.08 | 0.27 | 51.5 | 5.09 | 7 | 15% |
| 3868 | P08779 | Keratin 16; KRT16 ^§^ | 0 | 0.45 * | 51.1 | 4.98 | 5 | 11% |
| 3872 | NP_000413.1 | Keratin 17; KRT17 ^§^ | 0 | 0.27 * | 48 | 4.97 | 2 | 4% |
| 85288 | CAC27577 | Keratin associated protein 4.6; KRTAP4-6 ^§^ | 0.08 | 0 | 9.7 | 4.53 | 1 | 11% |
| 8302 | P26718 | Killer cell lectin-like receptor subfamily C, member 4; NKG2-D type II integral membrane protein; KLRC4 ^§^ | 0.15 | 0 | 25.3 | 8.56 | 1 | 4% |
| 3938 | S01168 | Lactase (EC 3.2.1.108) and glycosylceramidase (EC 3.2.1.62) complex; LCT ^§^ | 0.08 | 0 | 218.6 | 5.9 | 1 | 1% |
| 10288 | Q8N423 | Leukocyte immunoglobulin-like receptor, subfamily B (with TM and ITIM domains), member 2; LILRB2 ^§^ | 0.08 | 0 | 65.1 | 6.63 | 1 | 2% |
| 4046 | BAD96453.1 | Lymphocyte-specific protein 1; LSP1 ^§^ | 0.08 | 0 | 37.2 | 4.69 | 1 | 3% |
| 2217 | AAH08734 | MHC-related Fc receptor; neonatal transplacental IgG1/IgG3 transporter; FcRn; FCGRT ^§^ | 0.08 | 0 | 39.7 | 6.09 | 1 | 3% |
| 23499 | AAL38997 | Microtubule-actin crosslinking factor 1; macrophin 1 isoform 4; MACF1 ^§^ | 0.08 | 0 | 620.4 | 5.27 | 1 | 1% |
| 4133 | P11137 | Microtubule-associated protein 2; Alzheimer fibrils; MAP2^§^ | 0 | 0.18 | 199.5 | 4.83 | 1 | 1% |
| 84059 | NP_115495 | Monogenic, audiogenic seizure susceptibility 1 homolog (mouse); very large G-protein coupled receptor 1 (febrile seizures); MASS1 | 0.08 | 0 | 217.2 | 4.76 | 1 | 1% |
| 4585 | Q99102 | Mucin 4; MUC4 ^§^ | 0.08 | 0 | 58.7 | 4.79 | 1 | 2% |
| 4703 | P20929 | Nebulin; NEB ^§^ | 0 | 0.18 | 773.2 | 9.1 | 1 | 1% |
| 58157 | NP_067080 | Neuroglobin; NGB ^§^ | 0.08 | 0.18 | 16.5 | 5.42 | 2 | 16% |
| 4862 | NP_002509.2 | Neuronal PAS domain protein 2; seasonal affective disorder (SAD); NPAS2 ^§^ | 0.15 | 0.09 | 91.8 | 6.28 | 1 | 1% |
| 23529 | Q9UBD9 | Cardiotrophin-like cytokine factor 1; neurotrophin-1; B-cell stimulating factor-3 precursor; IL-6 superfamily; CLCF1 ^§^ | 0.08 | 0 | 25.2 | 8.68 | 1 | 3% |
| 5361 | CAB57274 | NOV/Plexin-A1; PLXNA1 ^§^ | 0 | 0.09 | 197.8 | 6.56 | 1 | 1% |
| 5004 | NM_000607 | Orosomucoid 1; α-1-acid glycoprotein 1; AGP 1; ORM1 | 0.15 | 0.36 | 23.5 | 5 | 11 | 36% |
| 5005 | CAI16860.1 | Orosomucoid 2; α-1-acid glycoprotein, type 2; ORM2 | 0 | 0.36 * | 23.5 | 5.01 | 5 | 23% |
| 5538 | CAI11025 | Palmitoyl-protein thioesterase 1; ceroid-lipofuscinosis, neuronal 1, infantile; PPT1 ^§^ | 0.08 | 0 | 34.2 | 6.07 | 1 | 3% |
| 5176 | AF400442 | Pigment epithelium-derived factor (PEDF); SERPINF1 | 0 | 0.45 * | 36.8 | 5.84 | 3 | 12% |
| 5730 | CAI12759 | Prostaglandin D2 synthase; PGD2S, 21kD, brain, β-trace protein = N-terminal 27 aa; PTGDS | 0.92 | 0.91 | 21 | 7.66 | 14 | 73% |
| 23272 | AAD55098 | Retinoblastoma-associated protein; RAP140 ^§^ | 0.08 | 0 | 140 | 5.46 | 1 | 1% |
| 6696 | BAC11635.1 | Secreted phosphoprotein 1; osteopontin-b, OPN; bone sialoprotein I; early T-lymphocyte activation 1; SPP1 | 0 | 0.27 * | 33.8 | 4.35 | 3 | 9% |
| 29106 | NP_037375.2 | Secretogranin III; SGG3 | 0 | 0.18 | 53 | 4.94 | 2 | 4% |
| 6493 | NP_033664 | Single-minded homolog 2 short isoform; SIM2 ^§^ | 0.08 | 0 | 73.2 | 9.03 | 1 | 2% |
| 11280 | Q9UI33 | Sodium channel, voltage-gated, type XI, α; voltage-gated sodium channel α subunit Nav1.9; sensory neuron sodium channel 2; SCN11A ^§^ | 0.15 | 0 | 204.9 | 8.32 | 2 | 1% |
| 79098 | NP_076427.1 | Specifically androgen-regulated protein; SARG; C1orf116 ^§^ | 0.08 | 0.09 | 64 | 8.78 | 1 | 2% |
| 10991 | Q99624 | System N amino acid transporter 1, SN1; N-system amino acid transporter 1; solute carrier family 38, member 3; SLC38A3 ^§^ | 0.08 | 0 | 55.8 | 8.03 | 1 | 2% |
| 7076 | P01033 | Tissue inhibitor of metalloproteinase 1; fibroblast collagenase inhibitor; erythroid potentiating activity; TIMP1 | 0 | 0.18 | 23.2 | 8.46 | 1 | 4% |
| 7273 | CAD12456 | Titin; TTN | 0 | 0.18 | 2993 | 6.35 | 3 | 1% |
| 2963 | P13984 | General transcription factor IIF, polypeptide 2, 30kDa, β subunit; TFIIF-β; transcription initiation factor RAP30; GTF2F2 ^§^ | 0 | 0.18 | 28.4 | 9.24 | 2 | 8% |
| 7018 | P02787 | Transferrin; TF | 0.69 | 0.91 | 77 | 6.81 | 55 | 54% |
| 7276 | P02766 | Transthyretin; prealbumin, amyloidosis type I; TTR | 0.77 | 0.91 | 12.8 | 5.33 | 11 | 100% |
| 7277 | P68366 | Tubulin α-1; testis-specific; TUBA1 | 0.39 | 0.18 | 49.9 | 4.95 | 5 | 11% |
| 80086 | NP_079295 | Tubulin α-4; TUBA4 ^§^ | 0.08 | 0 | 50.2 | 4.94 | 1 | 2% |
| 81027 | Q9H4B7 | Tubulin β-1; TUBB1 ^§^ | 0.08 | 0 | 50.3 | 5.05 | 3 | 6% |
| 7280 | P07437 | Tubulin β-2; TUBB2 | 0.23 | 0.09 | 49.7 | 4.78 | 1 | 2% |
| 10381 | Q13509 | Tubulin β-3; TUBB3 ^§^ | 0.08 | 0 | 50.4 | 4.83 | 1 | 2% |
| 27175 | Q9NRH3 | Tubulin γ-; TUBG2 ^§^ | 0 | 0.18 | 51.5 | 5.5 | 2 | 4% |
| 5799 | BQ787268 | Tyrosine phosphatase, receptor type, N2 (EC 3.1.3.48); PTPRN2 | 0.08 | 0 | 111.3 | 5.55 | 1 | 1% |
| 11041 | O43505 | UDP-GlcNAc:β-Gal-β-1,3-N-acetylglucosaminyltransferase 6; B3GNT6 ^§^ | 0 | 0.18 | 47.1 | 6.77 | 1 | 2% |
| 563 | P25311 | Zinc-α-2-glycoprotein; AZGP1 | 0 | 0.18 | 33.9 | 5.57 | 4 | 13% |
| 12 | P01011 | α-1-Antichymotrypsin; SERPINA3 | 0.08 | 0.45 * | 46.6 | 5.37 | 4 | 9% |
| 5265 | P01009 | α-1-Antitrypsin; SERPINA1 | 0.54 | 0.82 | 46.6 | 5.37 | 21 | 45% |
| 1 | P04217 | α-1-B-glycoprotein; A1BG | 0 | 0.18 | 31.9 | 5.65 | 5 | 17% |
| 2 | P01023 | α-2-Macroglobulin; A2M | 0 | 0.36 * | 163.3 | 5.95 | 11 | 11% |
| 567 | P01884 | β-2-Microglobulin; B2M | 0.15 | 0.18 | 13.8 | 5.77 | 3 | 24% |
